# Anti-tumor effects of fibroblast growth factor-binding protein (FGF-BP) knockdown in colon carcinoma

**DOI:** 10.1186/1476-4598-10-144

**Published:** 2011-11-23

**Authors:** Daniel Schulze, Philipp Plohmann, Sabrina Höbel, Achim Aigner

**Affiliations:** 1 Institute of Pharmacology, Faculty of Medicine, Philipps-University Marburg, Germany; 2 Rudolf-Boehm-Institute of Pharmacology and Toxicology, Clinical Pharmacology, University of Leipzig, Germany

**Keywords:** FGF-BP, RNAi, apoptosis, siRNA, polyethylenimine, PEI, colon carcinoma, gene targeting

## Abstract

**Background:**

Fibroblast growth factors FGF-1 and FGF-2 are often upregulated in tumors, but tightly bound to heparan sulphate proteoglycans of the extracellular matrix (ECM). One mechanism of their bioactivation relies on the FGF-binding protein (FGF-BP) which, upon reversible binding to FGF-1 or -2, leads to their release from the ECM. FGF-BP increases tumorigenicity and is highly expressed in tumors like colon carcinoma. In this paper, we analyse cellular and molecular consequences of RNAi-mediated FGF-BP knockdown in colon carcinoma, and explore the therapeutic effects of the nanoparticle-mediated delivery of small interfering RNAs (siRNAs) for FGF-BP targeting.

**Results:**

Employing stable RNAi cells, we establish a dose-dependence of cell proliferation on FGF-BP expression levels. Decreased proliferation is mirrored by alterations in cell cycle distribution and upregulation of p21, which is relevant for mediating FGF-BP effects. While inhibition of proliferation is mainly associated with reduced Akt and increased GSK3β activation, antibody array-based analyses also reveal other alterations in MAPK signalling. Additionally, we demonstrate induction of apoptosis, mediated through caspase-3/7 activation, and alterations in redox status upon FGF-BP knockdown. These effects are based on the upregulation of Bad, Bax and HIF-1α, and the downregulation of catalase. In a therapeutic FGF-BP knockdown approach based on RNAi, we employ polymer-based nanoparticles for the in vivo delivery of siRNAs into established wildtype colon carcinoma xenografts. We show that the systemic treatment of mice leads to the inhibition of tumor growth based on FGF-BP knockdown.

**Conclusions:**

FGF-BP is integrated in a complex network of cytoprotective effects, and represents a promising therapeutic target for RNAi-based knockdown approaches.

## Background

Fibroblast growth factors (FGF) represent a large polypeptide growth factors family comprising at least 23 members. Beyond embryonic development and tissue repair in the adult, FGFs play important roles in cancer and other diseases (see e.g. [1] for review). FGF-1 (acidic FGF, aFGF) and FGF-2 (basic FGF, bFGF) are the best-studied members and are often upregulated in tumors. Since both are tightly bound to heparan sulphate proteoglycans of the extracellular matrix (ECM), their bioactivation in terms of release from the ECM is required in order to allow their binding to FGF-receptors. While tissue destruction or the digestion of the HSPG sugar backbones by heparinases or other degrading enzymes may lead to enhanced FGF release under certain circumstances, another mechanism relies on an FGF-binding protein, FGF-BP1, acting as a chaperone molecule for FGFs.

FGF-BP was first isolated from the supernatants of A431 epidermoid carcinoma cells and termed HBp17 [2]. It is able to reversibly bind to FGF-1, -2, -7, -10, and -22 [2-6], leading to reduced heparin affinity e.g. of FGF-2 which is thus released from the extracellular matrix [3,7,8]. FGF-BP is highly expressed in some organs during embryonic development, but rapidly downregulated thereafter [9]. In adult tissues, FGF-BP is expressed during wound healing and in carcinogenesis. Upregulation in carcinogenesis occurs already at early stages of malignant transformation and is maintained throughout development into invasive carcinoma [9-13]. In fact, several studies have demonstrated FGF-BP overexpression in various tumors and tumor cell lines including HNSCC, melanoma, cervix, prostate, mamma, pancreatic and colon carcinoma [13-17]. Upregulation of FGF-BP can occur, among others, by TPA through Krüppel-like factor 5 (KLF-5) [18,19], DMBA [11], Wnt/β-catenin signalling [12], HPV16 E6 [20], androgen receptor activation [21] or EGF [22], while FGF-BP downregulation has been described for retinoids [23-25], TGF-β [26] or p53wt overexpression [27].

Supporting the functional relevance of FGF-BP in tumors, its overexpression was shown to increase tumorigenicity of FGF-BP-negative SW-13 cells, leading to the formation of highly vascularized tumors in immunodeficient mice [16,28]. Induction of angiogenesis was also demonstrated in a chorioallantoic membrane assay [3]. Concomitantly, ribozyme-mediated depletion of FGF-BP led to reduced tumor growth and decreased angiogenesis in SCC or prostate carcinoma cell lines [15,29]. Taken together, these results established FGF-BP as rate-limiting in tumor growth and as an 'angiogenic switch molecule' (see [30] for review). While FGF-BP exerts tumor-promoting effects through the activation of FGF-2 and activates FGF-2 [3,7], this does not exclude additional functions other than enhancing FGF activity, as suggested e.g. by the presence of FGF-BP in the nucleus (Aigner et al., unpublished data).

In colon carcinoma, FGF-BP has been shown to be upregulated in early dysplastic lesions of the human colon as well as in primary and metastatic colorectal cancers [12,13,29]. Stably ribozyme-transfected cells indicated reduced tumor growth upon FGF-BP knockdown and an inhibitory antibody led to reduced cell proliferation in vitro [12,13,29].

In this paper, we identify several cellular and molecular consequences of RNAi-mediated FGF-BP knockdown in colon carcinoma, and demonstrate that FGF-BP is integrated in a complex network of cytoprotective and proliferative effects. From these data and in vivo treatment studies with polymeric nanoparticles for siRNA delivery in s.c. colon carcinoma xenograft-bearing nude mice, we also conclude that FGF-BP represents a promising therapeutic target, and establish RNAi-based knockdown approaches through delivery of therapeutic siRNAs for FGF-BP inhibition.

## Methods

### ShRNA constructs and generation of plasmids

shRNA expression vectors were constructed by annealing synthetic complementary sense and antisense oligonucleotides containing siRNA sequences against hFGF-BP1 (NM_005130), a 9 nucleotide hairpin loop sequence (TTCAAGAGA) and a RNA Pol III terminator sequence tract (TTTTTT), flanked by a *XhoI *and a *HindIII *site. The sequences of the shRNA constructs were as follows:

FGF-BP "A" (mRNA 253-271):

5'GCACCCAGATTAAGCAGAAATTCAAGAGATTTCTGCTTAATCTGGGTGCTTTTTT-3'

FGF-BP "B" (mRNA 490-508):

5'GAGACATCTGTAGATATTCCTTCAAGAGAGGAATATCTACAGATGTCTCTTTTTT-3'

FGF-BP "C" (mRNA 364-382):

5'GGGTTGAGTGCACTCAATTGTTCAAGAGACAATTGAGTGCACTCAACCCTTTTTT-3'

Firefly Luciferase shRNA (humanized, pGL3):

5'GTGCGCTGCTGGTGCCAACTTCAAGAGAGTTGGCACCAGCAGCGCACTTTTTT-3'

The annealed oligonucleotides were cloned into the pSingle-tTS-Vector (Clontech) and the plasmids were checked by sequencing for correct insertion. The luc3 shRNA and the empty vector were used as negative controls.

### Generation of stable mass-transfected and clonal cell lines

LS174T, HCT-116 and HT29 colon carcinoma cells were obtained from the American Type Culture Collection (ATCC, Manassas, VA), HCT-116 p21 -/- were obtained from Dr. Bert Vogelstein [31], and stable FGF-BP expressing SW-13 adrenal carcinoma cells have been described previously [16]. Cells were cultivated under standard conditions (37°C, 5% CO_2_) in Iscove's modified Dulbecco's medium (PAA Laboratories, Cölbe, Germany) supplemented with 10% fetal calf serum (FCS) unless indicated otherwise.

Stable transfections were performed in 60 mm Petri dishes with 500,000 LS174T cells and 5 μg plasmid DNA using TurboFect (Fermentas, St. Leon-Rot) or 200,000 cells (HCT-116, HT29) and 2 μg plasmid DNA using Fugene (Promega, Mannheim, Germany) as described by the manufacturers. Selection of stable integrants was started 48 hours after transfection by adding 750 μg/ml (LS174T) or 1000 μg/ml (HCT-116, HCT-29) G418 (PAA) for 2 weeks. In the case of HCT-116 p21-/- cells, 0.2 μg pcDNA 3.1/Hygro (Life Technologies, Darmstadt, Germany) was added to the transfection, and stable cell lines were selected in the presence of 800 μg/ml hygromycin. Clonal selection from stably mass-transfected cells was performed by clonal expansion, and stably mass transfected cells or clonal shRNA expressing cell lines were used as indicated in the experiments.

### PCR and Quantitative RT-PCR (qRT-PCR)

The stable integration of the shRNA expression cassette was confirmed by PCR analysis of genomic DNA with vector specific primers (Sing_TetU6) using the High-Pure PCR Template Preparation Kit according to the manufacturer's protocol (Roche Diagnostics, Mannheim, Germany).

Total RNA from tumor xenografts or cells from tissue culture was isolated using the TRI reagent (Sigma, Deisenhofen, Germany) according to the manufacturer's protocol. Reverse transcription (RT) was performed using the RevertAid H Minus First Strand cDNA Kit (Fermentas) with 1 μg total RNA and random hexamer primers. Quantitative polymerase chain reaction was performed using a LightCycler (Roche) in a total volume of 10 μl per capillary containing 5 μl QuantiTect SYBR Green 2× Master Mix, 4 μl diluted cDNA (1:50) and 1 μl intron spanning qPCR primers (5 μM each) specific for FGF-BP or reference genes (β-actin, GAPDH). The Hot Start Polymerase was activated by a 15 min pre-incubation at 95°C, followed by 55 amplification cycles at 95°C for 10 seconds, 55°C for 10 seconds and 72°C for 10 seconds. CP values obtained were between 22 and 25 for FGF-BP and between 15 and 16.5 for actin/GAPDH. A melting curve analysis was performed to verify correct PCR products and a sample containing no template was always run in parallel to control for background, which was at CP values > 50. Quantitation of gene expression was performed by the ΔΔC_t _method with β-actin and GAPDH serving as reference housekeeping genes.

### Western blot analysis

To determine the RNAi-mediated downregulation of FGF-BP at the protein level, LS174T cells from cell culture were scraped and lysed in PBS/5 mM EDTA in presence of Protease Inhibitors ('lysis buffer') on ice, or tumor xenograft tissue was homogenized in liquid nitrogen, and upon addition of lysis buffer treated by sonication or three freeze-thaw cycles. After centrifugation and determination of the protein concentration in the supernatant, samples containing 100 μg protein were separated by SDS-PAGE (19% polyacrylamide containing 6 M Urea and a 10% Spacer Gel) and transferred onto a nitrocellulose membrane (Protran Nitrocellulose Transfer Membrane 0,45 μm Schleicher&Schuell, Dassel, Germany) by semi-dry blotting. Membranes were blocked with 5% milk powder in Tris-buffered saline + Tween (TBST; 20 mM Tris, pH 7.5, 150 mM NaCl, and 0.1% Tween-20) and incubated with either of the following antibodies: 1:1000 rabbit polyclonal anti-FGF-BP raised against a GST-human FGF-BP fusion protein [23,29], 1:5000 anti-HIF1α (Epitomics, Burlingame, CA), 1:2000 anti-catalase (Epitomics), 1:500 anti-p21 (Santa Cruz, CA, USA), 1:250 anti-phosphoAkt (Santa Cruz) or 1:5000 anti-β-actin antibodies (Santa Cruz). The blots were then washed in TBST and incubated with a donkey anti-rabbit secondary antibody, diluted 1:2000 in TBST, coupled to horseradish peroxidase (GE Healthcare Bio-Sciences, Uppsala, Sweden) for 1 h at RT. After washing in TBST, bound antibodies were visualized by chemiluminescence (ECL SuperSignal West Pico Kit, Thermo Fisher Scientific, Rockford, IL, USA).

### In vitro Proliferation and Growth Assays

Soft agar assays for the determination of anchorage-independent proliferation and colony formation were carried out essentially as described previously [32]. Briefly, 20.000 cells in 0.8 ml 0.35% agarose (Bacto Agar, Becton Dickinson, Franklin Lakes, NJ) were layered on top of 1 ml solidified 0.6% agar in a 6-Well plate. IMDM/10% FCS was included in both layers. After 2 week, colonies > 50 μm were counted by at least two independent investigators blinded to the study. Anchorage-dependent proliferation of stable cells lines was studied in triplicates using a WST-1 colorimetric assay according to manufacturer's protocol (Cell proliferation Reagent WST-1, Roche) and as described previously [33]. Briefly, cells were seeded into 96-Wells at 500 to 1000 cells per well and the proliferation rate was assessed by determining the number of living cells at each time point. For GSK3β inhibition, a 10 mM 6-bromoindirubin-3-oxime (BIO; Sigma, Deisenhofen, Germany) stock solution in DMSO was diluted in medium as indicated in the figure and added to the cells. For FGF2 stimulation, recombinant FGF2 (NatuTec, Frankfurt, Germany) from a 100 μg/ml stock solution was added to the medium at the concentrations indicated in the figure. Transient siRNA transfections in 96-wells were performed using INTERFERin (PEQLAB, Erlangen, Germany) and 1 pmol siRNA/well, prior to the WST-1-based measurement of viable cells at the time points indicated.

### Apoptosis assays

To test for apoptosis *in vitro*, a commercially available bioluminescent caspase-3/7 assay (Caspase-Glo^® ^3/7 assay, Promega, Mannheim, Germany) and a fluorometric Assay based on the caspase-3 substrate Ac-DEVD-AMC (Alexis) were applied. The Caspase-Glo 3/7 assay was performed in the 96-well format as recommended by the supplier and luminescence was measured after 1 hour incubation at 27°C in the dark using a Fluostar Optima reader (BMG Labtec, Jena, Germany). To normalize for differences in cell densities, a WST-1 assay was performed in parallel on the same plate, and the results of caspase activity were adjusted to cell numbers of the different cell lines.

The Caspase-3 assay using Ac-DEVD-AMC was assessed in a 6-Well format at a density of 100.000 cells. At the time points indicated in the figures, cells were washed twice with PBS and scraped from the surface. Cell lysis was performed in 300 μl lysis buffer (50 mM Tris/HCl pH 7.4, 150 mM NaCl, 2 mM MgCl_2_, 10 mM DTT and Protease Inhibitor (Protease Inhibitor Cocktail Set III, Calbiochem)) followed by sonication in an ultrasonic bath three times for 10 sec. and chilling on ice. Cellular debris was removed by centrifugation at 10,000 *g *for 5 min and the total protein content of the supernatant was determined by a Bio-Rad protein assay. 30-100 μg total protein was diluted in 40 μl of freshly prepared reaction buffer containing PBS/10 mM DTT. 40 μl supernatant was transferred to 96 well plates (white walled), the reaction was started by adding 10 μl substrate solution (10 mM Ac-DEVD-AMC (Acetyl-Asp-Glu-Val-Asp-7-Amino-4-methylcoumarin) stock solution in DMSO, dissolved in 500 μl PBS) to a final concentration of 25 μM Ac-DEVD-AMC and measured using a FLUOstar OPTIMA microplate reader (λ_ex _= 380 nm, λ_em _= 475 nm). A background control containing the substrate in PBS/10 mM DTT without cell lysate was run in parallel and a time curve of fluorescence was measured at 37°C every 20 min to check for linearity. Averages of triplicates were subtracted from the background signal and values were normalized as x-fold increase in apoptosis rate relative to the corresponding values of control cells at time points indicated in the figures. Cells treated with 50 μg/ml 5-fluorouracil served as positive control.

### Analysis of differential gene expression by Phospho-MAP Kinase and Apoptosis Antibody Array Kits

The activity of a panel of mitogen-activated protein kinases (MAPKs) and other serine/threonine kinases was assessed through the determination of their relative levels of phosphorylation using the Proteome Profiler Array (R&D Systems, Wiesbaden, Germany) according to the manufacturer's instructions. Briefly, stable FGF-BP depleted cells and control cells were seeded at 50% confluency in 6-well plates and grown for 2 days prior to lysis at 1 × 10^7 ^cells/ml in lysis buffer for 30 minutes at 4°C. After centrifugation at 14,000 *g *for 5 minutes, the protein concentration of the supernatant was determined, and the volume of each sample equivalent to 250 μg of protein was diluted in Array Buffer 1 to yield a final volume of 1.5 ml. Arrays were pre-incubated in 1.5 ml Array Buffer 1 for 1 hour before incubating the array strips in the diluted sample at 4°C overnight, washing 3 × 10 minutes in 20 ml wash buffer, incubating in the detection antibody cocktail (1:100 in 1X Array Buffer 1), washing, and incubating in a Streptavidin-HRP solution (1:2000). After washing again, signals were developed by incubating in a chemiluminescent substrate (SuperSignal West Femto Kit, Thermo Fisher Scientific, Rockford, IL), and chemiluminescence was visualized by film exposure (Hyperfilm ECL; GE Healthcare, Munich, Germany). To precisely analyze very strong as well as very weak signals within the linear range, exposure times were varied between 5 seconds and 2 minutes. Signals were scanned and quantitated by densiometry using ImageJ (National Institutes of Health, Bethesda, MD).

### Analysis of cell cycle distribution

For flow cytometry-based analysis of cell cycle distribution, the sample preparation and propidium iodide staining of nuclear DNA were performed as described previously [34]. Briefly, 200.000 cells were grown at ~50% confluency, harvested by trypsinization, washed twice with PBS and fixed in 70% ethanol diluted in PBS at -20°C for 1 h. The cells were resuspended in PBS and incubated with 100 μg/ml RNAse in PBS at 4°C overnight, prior to addition of 40 μg/ml propidium iodide and analysis by flow cytometry. To monitor FGF-BP-dependent effects on cell cycle, cells were arrested in the G2/M phase by pre-treatment with 250 ng/ml nocodazole (750 nM) for 24 h. The cells were then either harvested and processed as described above, or washed twice with PBS and further cultivated in fresh medium for another 24 h to release the nocodazole-induced mitotic cell cycle arrest prior to cell cycle analysis. Distribution of cell cycle was determined using a FACS Calibur (Becton-Dickinson) with an argon laser set to excite at 488 nm and measuring FSC, SSC, peak width and area of fluorescence. Counts were gated to exclude aggregates and subcellular debris, and from a minimum of 20,000 gated events for each sample, a frequency histogram of peak areas was generated and analysed using Cell-Quest software.

### Subcutaneous tumor xenograft model in nude mice

Effects of RNAi-mediated FGF-BP knockdown on LS174T tumor growth *in vivo *was determined by treating subcutaneous tumor xenograft-bearing mice with siRNAs complexed with polyethylenimine (PEI) as described previously [35]. 5 × 10^6 ^LS174T wildtype cells were injected subcutaneously into both flanks of athymic nude mice (Crl:CD1-Foxn1nu, Charles River Laboratories, Sulzfeld, Germany). When solid tumors were established after 5 days, mice were randomized into treatment or control groups with 6-8 animals per group. Mice in the specific treatment group were injected intraperitoneally (i.p.) with 0.77 nmol (10 μg) FGF-BP-specific siRNA duplexes (5'-GCCAGAGUGAUAAUUUCAGTT-3'/5'-CUGAA AUUAUCACUCUGGCTC-3'), complexed with PEI F25-LMW as described previously [35,36], every 3 times per week for 4 weeks. PEI F25-LMW-complexed non-specific siRNA (pGL3; 5'-CGUACGCGGAAUACUUCGATT-3'/5'-UCGAAGUAUUCCGCGUACGTT-3'), injected in the same manner, as well as untreated mice served as negative controls. Tumor volumes were measured three times per week as indicated in the figure. Upon termination of the experiment, mice were sacrificed and tumors were removed. Pieces of each s.c. tumor xenograft were immediately shock frozen for preparation of RNA or of protein.

For the radioactive determination of siRNA delivery into the tumor, 0.6 μg (0.05 nmoles) FGF-BP-specific siRNAs were [^32^P] end-labeled at both strands using T 4 polynucleotide kinase and γ-[^32^P] ATP. To remove unbound radioactivity, siRNAs were purified by microspin columns (Bio-Rad, München, Germany) and complexed prior to i.p. injection as described above. After 2 h, mice were sacrificed and tumors were removed for RNA preparation as described above. The total RNA was dissolved in 200 μl DEPC-treated water, and 10 μl-samples were mixed with loading buffer, heat-denatured and subjected to agarose gel electrophoresis prior to blotting and autoradiography (Biomax, Eastman-Kodak, Rochester, NY). Quantitation was performed by phosphor imager analysis.

Animal studies were conducted according to guidelines of animal welfare and approved by the Regierungspräsidium Giessen.

### Statistics

Statistical analyses were performed by Student's t-test, One-way ANOVA/Tukey's multiple comparison post-tests or Two-way ANOVA using GraphPad Prism4, and significance levels are * = p < 0.05, ** = p < 0.01, *** = p < 0.001, # = not significant. Values are shown +/- s.e.m.

## Results

### RNAi-mediated FGF-BP knockdown exerts 'gene dose-dependent' anti-proliferative effects in colon carcinoma cells in vitro

**Figure 1:**
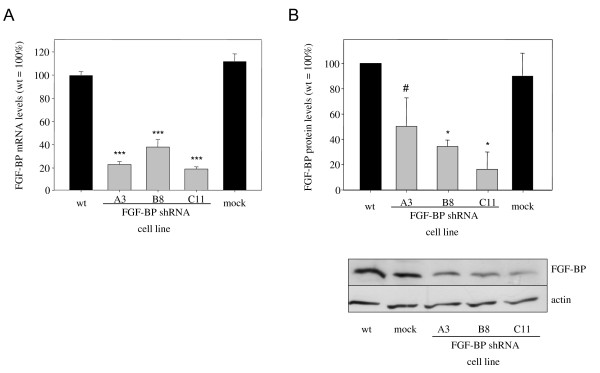
**RNAi-mediated knockdown of FGF-BP in LS174T colon carcinoma cells**. LS174T cells stably mass-transfected with shRNA expression plasmids and clonally selected show reduced FGF-BP expression as determined on mRNA (qRT-PCR, A) and protein level (Western blot, B).

LS174T cells were stably mass transfected with shRNA expression plasmids and, upon generation of G418-resistant cells, clonal selection was performed through limited dilution. The analysis of FGF-BP expression levels in various clonal cell lines by qRT-PCR demonstrated the functionality of all three shRNAs as compared to control-shRNA or non-transfected cells, with residual mRNA levels in clonal cell lines being between 20% and > 90% (data not shown). For subsequent experiments, three clones (A3, B8, C11) were selected, which are based on the different shRNAs and showed stable knockdown efficacies of 60-80% (Figure 1A). The parallel use of three clonal cell lines stably transfected with different shRNAs, and the comparison between non-specific shRNA and wildtype cells, also allows to control for off-target effects specifically based on a certain sequence (see e.g. [37] for review) or non-specifically based on increased shRNA expression [38]. Knockdown efficacies in the stable cell lines were confirmed on protein levels by Western blotting and revealed an RNAi-mediated reduction of FGF-BP protein by ~ 50% (A3) to > 80% (C11) (Figure 1B).

**Figure 2:**
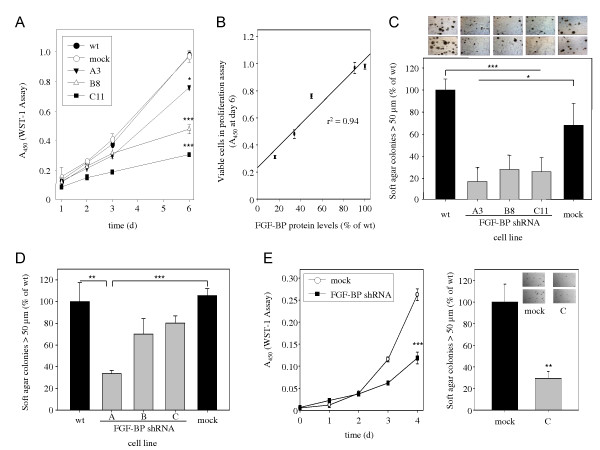
**Effects of FGF-BP expression on cell proliferation and colony formation**. Clonal or mass-transfected shRNA expressing LS174T cell lines with reduced FGF-BP levels show decreased anchorage-dependent cell proliferation as determined in WST-1 assays (A). Anti-proliferative effects in LS174T cells are dependent on the extent of RNAi-mediated FGF-BP knockdown, and the correlation between residual FGF-BP levels and the proliferative activity establishes an FGF-BP 'gene-dose effect' on cell proliferation (B). Soft-agar assays reveal marked reductions of anchorage-independent proliferation upon FGF-BP knockdown in LS-174T (C) and in HT29 cells (D). Anti-proliferative effects are confirmed in HCT-116 cells that show decreased anchorage-dependent (E, left) and anchorage-independent proliferation (E, right) upon FGF-BP knockdown.

When cells were analysed in proliferation assays, a significant reduction in anchorage-dependent proliferation was observed upon shRNA-mediated FGF-BP knockdown vs. negative controls (non-specific shRNA ('mock') transfected cells or wt cells). More specifically, the ~ 8-fold proliferation rate over 5 d in the control cells was reduced to ~ 5-fold upon 50% FGF-BP knockdown, to ~ 3-fold upon 60% FGF-BP knockdown, and to ~ 2-fold upon 80% knockdown (Figure 2A). Strikingly, the comparison between the different clonal cell lines also revealed that the anti-proliferative effects were directly correlated with residual FGF-BP protein levels (Figure 2B; r^2 ^= 0.94). Thus, this establishes an FGF-BP gene dose effect on LS174T cell proliferation, further supporting the functional relevance of FGF-BP on LS174T colon carcinoma cell growth.

The anti-proliferative effects of FGF-BP knockdown were confirmed in soft-agar assays, which monitor the anchorage-independent growth and thus resemble more closely the in vivo situation. Here, however, the 50% FGF-BP knockdown resulted already in a substantial > 70% reduction in colony formation over wt control cells (with negative control shRNA-transfected cells showing a slight reduction in colony numbers as well), with no further decrease in colony formation being observed in the cell lines with lower FGF-BP levels (Figure 2C). More specifically, FGF-BP knockdown, independent of residual FGF-BP levels, resulted in smaller-sized colonies and smaller colony numbers (Figure 2C, upper panel). This suggests that LS174T cells growing under anchorage-independent conditions are even more dependent on FGF-BP expression than when cultivated on plastic, and further emphasizes the possible relevance of FGF-BP as therapeutic target for knockdown approaches in vivo.

The rate-limiting effect of FGF-BP on cell growth was further confirmed in other colon carcinoma cell lines. FGF-BP knockdown in HT29 cells revealed a ~ 20-60% reduction of soft agar colony formation (Figure 2D). Likewise, profound > 50% antiproliferative effects were observed in anchorage-dependent proliferation (Figure 2E, left) and anchorage-independent soft agar colony formation upon transfection of HCT-116 cells with FGF-BP shRNA (Figure 2E, right).

### FGF-BP knockdown leads to alterations in cell cycle and induction of apoptosis

**Figure 3:**
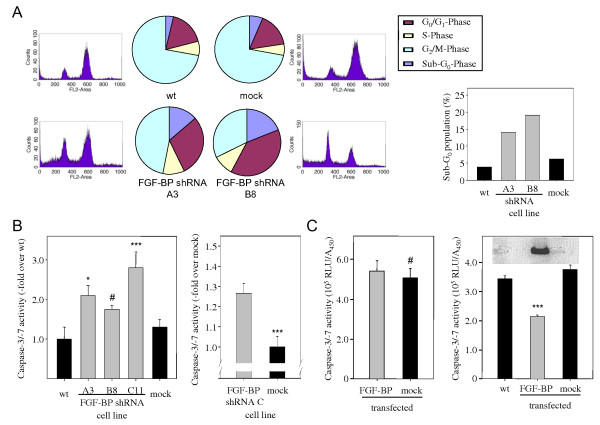
**Effects of FGF-BP expression on cell cycle and apoptosis**. Stable FGF-BP knockdown cells vs. control LS174T cells were treated with nocodazole 20 h prior to propidium iodide/FACS cell cycle analysis. Decreased percentages of cells in G2/M arrest demonstrate slower cell cycle progression after FGF-BP knockdown (A), while sub-G0 levels are increased (A; see also right bar diagram). Caspase-3/7 assays show the induction of apoptosis, represented by increased caspase-3/7 activity, upon FGF-BP knockdown in LS174 (B, left) or HCT-116 cells (B, right). Expression of exogenous FGF-BP leads to anti-apoptotic effects in endogenously FGF-BP-negative SW13 cells (C, right), but not in LS174T cells with already high endogenous FGF-BP levels (C, left).

To analyse the effects of FGF-BP knockdown on LS174T colon carcinoma cell growth in more detail, the various cell lines were treated with nocodazole, a well-established compound for mediating G2/M arrest, 20 h prior to cell cycle analysis by propidium iodide staining and flow cytometry. In wt and negative control-transfected cells, the FACS-based cell cycle analysis revealed a profound nocodazole-mediated G2/M arrest (Figure 3, upper panel, left). In contrast, upon FGF-BP knockdown the nocodazole-mediated M-trapping was markedly reduced, indicating a slower cell cycle progression which results in a smaller fraction of cells being in the G2/M arrest after 20 h. Again, this effect was 'FGF-BP gene dose-dependent' with the deceleration in cell cycle progression being more profound in clone B8 vs. clone A3 cells (Figure 3A, lower panel). Notably, FGF-BP knockdown also resulted in a marked increase in the sub-G0 population, which is associated with apoptosis. Again, this effect was more profound in the B8 clone (Figure 3A, right).

To further analyse the effect of decreased FGF-BP expression on apoptosis, caspase activity was measured in the various stable cell lines in a caspase-3/7 assay. As compared to the negative control cells, a ~ 2-fold increase in caspase-3/7 activity was observed upon FGF-BP knockdown (Figure 3B, left). A slight but not significant trend towards higher apoptosis was observed in the clone C11 with the lowest FGF-BP levels. Likewise, a ~ 1.3 increase in apoptosis was observed in FGF-BP shRNA-transfected HCT-116 cells (Figure 3B, right). This establishes for the first time, and in contrast to previous results in other cell lines [15], that human FGF-BP exerts anti-apoptotic effects. For further analysis, we tested a reverse setting by overexpressing FGF-BP. In LS174T cells, no further reduction of apoptosis below levels in negative control cells was observed, indicating that the forced expression of FGF-BP did not add an effect beyond the anti-apoptotic effect of the FGF-BP (over-) expressed physiologically in this cell line (Figure 3C, left). This finding was independent of the cultivation conditions, i.e. the serum concentration in the medium (between 0% and 10%; data not shown). In contrast, the forced expression of FGF-BP in the adrenal carcinoma cell line SW-13, which is physiologically FGF-BP-negative, led to a significant ~ 40% reduction in the intrinsic apoptosis rate indicating an 'apoptosis rescue' upon FGF-BP overexpression (Figure 3C, right).

### Anti-proliferative effects of FGF-BP knockdown are based on alterations in phospho-MAPK status

**Figure 4:**
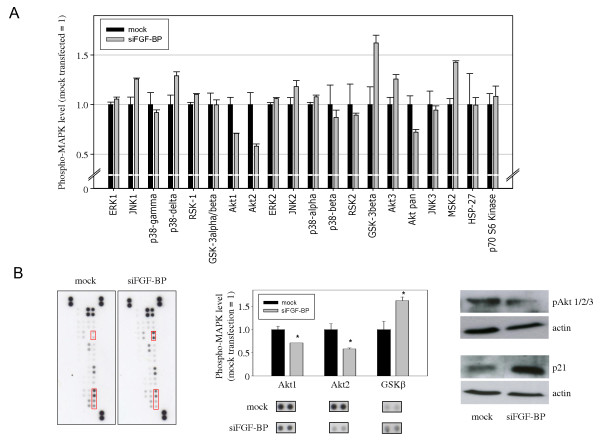
**Anti-proliferative effects of FGF-BP knockdown are based on alterations in phospho-MAPK status**. Phospho-MAPK Proteome Profiler antibody array probed with lysates from FGF-BP knockdown (grey bars) vs. mock-transfected (black bars) LS174T cells (A). Original blots with Akt1, Akt2 and GSK3β signals being highlighted by red frames (B, left); observed differences are statistically significant (B, center). Results from antibody arrays are confirmed in independent experiments by Western blotting for Akt (B, upper right) and p21 (B, lower right; see Figure 5 for array).

To analyse the anti-proliferative effects of FGF-BP inhibition in more detail on the molecular level, the activity of various downstream signal transduction molecules was monitored through the determination of their respective phosphorylation levels in a Phospho-MAPK antibody array. Signal intensities in negative control-transfected ('mock transfected') cell lysates were compared to lysates of the cells with the most profound knockdown, i.e. clone C11. Since signal intensities varied over a broad range dependent on the analyte dotted on the membrane, several exposures were scanned to ensure that only signals in the linear range were analysed. Upon FGF-BP knockdown, markedly decreased signal intensities were observed in Akt (pan), Akt1 and Akt2, indicating reduced Akt signal transduction (Figure 4A, B). Results were confirmed by Western blotting (Figure 4B, right panel). Notably, in contrast to previous publications [39] no changes in ERK activation were detected. On the other hand, activation upon FGF-BP knockdown was determined in the case of GSK3β and MSK2 and, to a lesser extent, in JNK (Figure 4A, B). Other signal transduction molecules remained unchanged or showed no significant difference.

### Pro-apoptotic effects of FGF-BP knockdown are correlated with alterations in molecules involved in apoptosis and redox status

**Figure 5:**
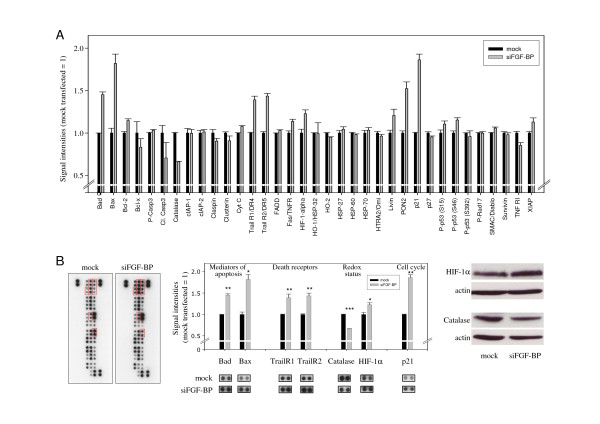
**Pro-apoptotic effects of FGF-BP knockdown are correlated with alterations in molecules involved in apoptosis and redox status**. Apoptosis Proteome Profiler antibody array probed with lysates from FGF-BP knockdown (grey bars) vs. mock-transfected (black bars) LS174T cells (A). Original blots, relevant signals are highlighted by red frames (B, left) and observed differences are statistically significant (B, center). Antibody array results were confirmed in independent experiments by Western blotting (B, right).

Since we found in this study an anti-apoptotic role of FGF-BP, FGF-BP knockdown was also analysed with regard to changes in molecules relevant in apoptosis. Indeed, upon FGF-BP reduction a marked activation of members of the bcl-2 family and promoters of apoptosis, Bad and Bax, and to a lesser extent of bcl-2, was observed in an apoptosis antibody array (Figure 5A, B). Furthermore, FGF-BP knockdown activated Trail receptors Trail R1 and R2. Regarding proteins related to the redox status of the cells, a significant inhibition of catalase and a moderate activation of HIF-1α was detected (Figure 5A, B). For the general confirmation of the robustness of the antibody array results, these findings were reproduced by Western blotting, indicating a similar inhibition of catalase and a somewhat more pronounced activation of HIF-1α (Figure 5B, right). Activation upon FGF-BP knockdown was also detected for livin and PON2, while signals specific for cleaved caspase-3 were reduced (Figure 5A). Finally, p21 showed ~2-fold higher levels after FGF-BP inhibition (Figure 5A, B; see also Figure 4B for Western blot confirmation), which is in line with the observed decrease in cell cycle progression (see Figure 3A).

### Analysis of cellular and molecular effects of FGF-BP knockdown

**Figure 6:**
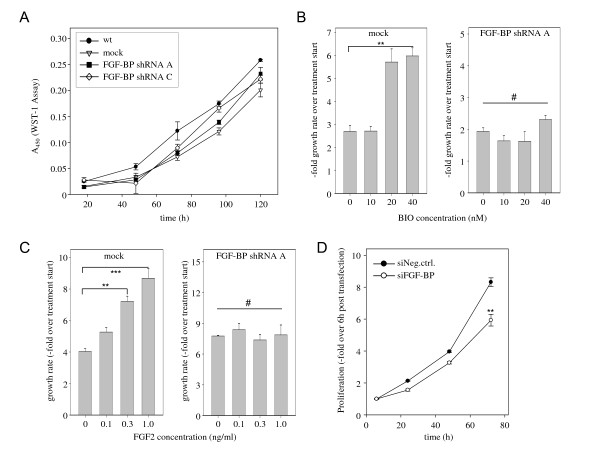
**Analysis of cellular and molecular effects of FGF-BP knockdown**. The effects of FGF-BP levels on cell proliferation are dependent on p21 as indicated by the absence of anti-proliferative effects of FGF-BP knockdown in HCT-116 p21 -/- cells (A). Dependence of antiproliferative GSK3β effects on FGF-BP. GSK3β inhibition by treatment of LS174T cells with 6-bromoindirubin-3-oxime (BIO) leads to the induction of proliferation (B, left). This effect is abrogated upon FGF-BP knockdown (B, right). Dependence of FGF2 cell proliferation-simulating effects on FGF-BP expression. Stimulation of LS174T cells by treatment with FGF2 leads to the induction of proliferation (C, left). This effect is largely abrogated upon FGF-BP knockdown (C, right). Anti-proliferative effects of transient siFGF-BP transfection (D).

To further analyse the role of downstream signal transduction pathways on the observed FGF-BP knockdown effects, stably FGF-BP shRNA-transfected p21-/- knockout cells were generated. In these cell lines, the proliferation rate was independent of FGF-BP expression levels (Figure 6A), which is in contrast to the p21 wt cells (see Figure 2E). This indicates that anti-proliferative effects of the FGF-BP knockdown may be based on the upregulation of p21 (see Figures 4B right, 5B center), thus being dependent on the presence of p21 expression.

Based on its role in various pathways including ERK1/2 activation, β-catenin signalling and c-Myc/cyclin D1 expression, previous studies have suggested a putative role of GSK3β as a tumor suppressor [40]. Interestingly, we observed that FGF-BP knockdown resulted in GSK3β upregulation (see Figure 4), prompting us to further study whether alterations in FGF-BP levels would influence effects of GSK3β inhibition. Indeed, while GSK3β inhibition by treatment of LS174T cells with 20 nM or 40 nM 6-bromoindirubin-3-oxime (BIO) led to a > 2-fold induction of cell proliferation (Figure 6B, left), this effect was largely lost upon FGF-BP knockdown (Figure 6B, right). Here, a statistically non-significant increase in cell proliferation was only observed at 40 nM BIO, suggesting that the GSK3β upregulation upon FGF-BP knockdown may attenuate the effects of the inhibitor.

FGF-BP has been shown previously to exert its tumor-promoting role through the activation of FGF2, and to activate FGF2 [3,7]. To analyse whether FGF-BP influences FGF2-mediated stimulation of colon carcinoma cells, mock transfected or FGF-BP shRNA-transfected LS174T cells were treated with increasing amounts of FGF2. While a dose-dependent stimulation of cell proliferation was observed in the cells with high endogenous FGF-BP expression (Figure 6C, left), this effect was completely abrogated after FGF-BP knockdown (Figure 6C, right). This indicates that the cellular effects observed upon FGF-BP knockdown are, at least in part, due to a reduction in FGF2-mediated stimulation.

Finally, we analysed if tumor cell inhibition is also obtained after a transient siRNA-mediated FGF-BP knockdown, thus avoiding the generation of stable cell lines with possible adaptation processes during the selection procedure. Indeed, a statistically significant reduction in HT29 cell proliferation was observed (Figure 6D). This also provided the basis for using siRNAs in a therapeutic in vivo approach.

### Anti-tumor effects of therapeutic FGF-BP knockdown in vivo

**Figure 7:**
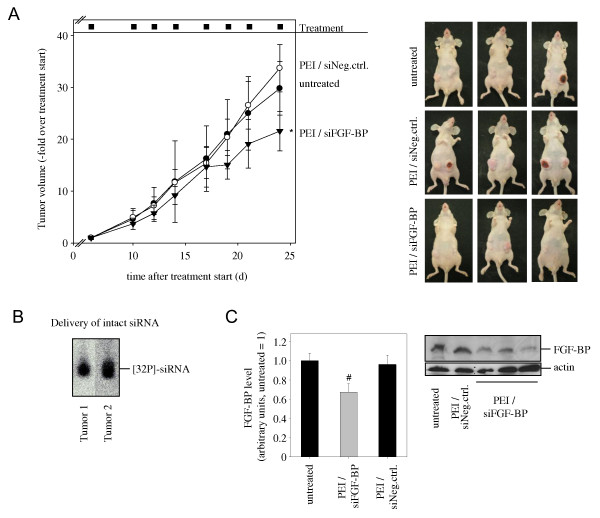
**Anti-tumor effects of therapeutic FGF-BP knockdown in vivo**. Upon establishment of s.c. LS174T tumor xenografts, tumor-bearing mice were randomized and treated at the indicated time points by systemic injection of 10 μg PEI-complexed siRNAs. PEI/siRNA-mediated knockdown leads to tumor growth inhibition reaching statistical significance between PEI/siFGF-BP and PEI/siNeg.ctrl. (A, left). Termination of the experiment upon occurrence of mechanical wounds in the tumors, resulting in the loss of tumor mass in some mice of the control-treated and untreated groups (A, right for representative examples of mice). Oberserved anti-tumor effects are based on the PEI-mediated delivery of intact siRNAs as determined by [32P]-labeled siRNA molecules (B), leading to reduced FGF-BP protein levels in the treatment group as determined by Western blotting (C; see right panel for representative examples).

To assess the therapeutic relevance of FGF-BP as a target for gene knockdown approaches, we employed a polyethylenimine (PEI)-based delivery platform for siRNAs previously established in our lab [35,41] in order to induce RNAi in already established tumors. Subcutaneous tumor xenografts were generated by injecting wt LS174T tumor cells and, upon formation of solid tumors, mice were randomized and treated systemically through intraperitoneal (i.p.) injection of PEI/siRNA complexes. I.p. administration was preferred over i.v. injection due to the more efficient siRNA delivery [35]. Although LS174T had been found to be rather hard to transfect with PEI complexes in vitro (data not shown), the analysis of the levels of [32P]-labeled, PEI complexed siRNAs in vivo revealed the delivery of intact siRNAs into the tumor (Figure 7B). For therapeutic intervention, mice with established tumor xenografts were treated three times per week as indicated in Figure 7A with PEI-complexed, FGF-BP-specific siRNAs. No treatment or treatment with PEI-complexed, non-specific siRNAs at the same time points served as negative controls. Upon termination of the experiment after 3 weeks of treatment, a ~ 40% reduced tumor growth was observed in the FGF-BP-specific knockdown group (Figure 7A) as compared to the negative control treatment. Due to their size, some tumors in the no treatment and in the negative control treatment groups showed poor tissue integrity with some wounding and a partial loss of tumor mass already prior to the time point of termination of the experiment, which may rather lead to a slight under-estimation of PEI/siRNA-mediated antitumor effects (see Figure 7A, right, for some representative examples). Concomitant with the observed reduction in tumor growth, Western blotting of the tumor lysates that were available for analysis revealed ~ 30% reduced FGF-BP levels in the tumor xenografts of the specific treatment group as compared to the controls, which both showed identical levels (Figure 7C). From these data we conclude that already a rather moderate ~ 30% knockdown of FGF-BP exerts anti-tumor effects.

## Discussion

In this paper, we show that FGF-BP knockdown exerts tumor-inhibiting effects in colon carcinoma in vitro and in vivo, which are based on anti-proliferative as well as pro-apoptotic effects in tumor cells. Our cell cycle experiments demonstrate that anti-proliferative effects rely on a G0/G1 arrest leading to cell cycle prolongation. On a molecular level, this involves the cell cycle control protein p21^WAF1/CIP ^which is upregulated upon FGF-BP knockdown. Usually, p21 acts as tumor suppressor that is p53-dependently upregulated upon genotoxic effectors or cellular stress [42]. Interestingly, in our system the induction of p21 was independent of p53, since no changes in p53 activity (phosphorylation) were observed although LS174T cells are p53 (+/+) [43]. This mechanism of p53-independent induction of p21 has been described previously (see e.g. [44]). The relevance of p21 in mediating FGF-BP effects on proliferation is supported by the abrogation of inhibitory effects of an FGF-BP knockdown on the proliferation of p21 knockout cells shown here. Furthermore, FGF-BP has been demonstrated to be involved in the cellular signal transduction, leading to FGF-2 induced phosphorylation of ERK1/2 [3] and Akt [45]. It was also shown that the overexpression of the positive FGF-BP regulator KLF-5 leads to the activation of Akt kinases [46], which have been described in several studies as relevant in colon carcinoma tumorigenesis (see e.g. [47]). In cellular survival signalling, Akt kinases play a pivotal role by (i) blocking pro-apoptotic proteins [48], (ii) inhibiting the SAPK/JNK pathway [49] and (iii) antagonizing p21 induction (see [50] for review). Indeed, upon FGF-BP knockdown we observed Akt suppression and activation of p21 (see above), SAPK/JNK, caspases-3/7 and mediators of apoptosis, the cell death-enhancing *BH3-only domain *proteins Bad and Bax. Another Akt antagonist in PI3K/Akt signalling is GSK3, which is negatively regulated by Akt [51]. Concomitantly, we found that GSK3β is upregulated upon FGF-BP knockdown. This is also in line with the observation that cultivation of cells under serum-free conditions leads to increased GSK3 activity and apoptosis (see [52] for review). This GSK3β upregulation also led to a reduced sensitivity of FGF-BP shRNA-transfected cells towards the GSK3β inhibitor 6-bromoindirubin-3-oxime (BIO). While a recent study has described an attenuation of cell survival and proliferation upon GSK3β inhibition [53], our data rather support the notion of GSK3β acting as a tumor suppressor [40].

It has been established previously that FGF-BP knockdown leads to reduced bioactivation of FGFs from the ECM and thus lower effective concentrations. Indeed, we show FGF-BP knockdown was able to abolish the stimulatory effects of exogenous FGF2 in colon carcinoma cells. Although this confirms the role of FGF-BP in enhancing FGF activity, it does not exclude additional mechanisms of action, as suggested e.g. by the presence of FGF-BP in the nucleus of tumor cells (Aigner et al., unpublished data). While previous studies showed contradictory results whether FGF-BP enhances the anti-apoptotic effects of FGF-2 [45] or is not related to apoptosis [15], we clearly demonstrate in this paper the anti-apoptotic function of human FGF-BP in tumor cells. The fact that no additional anti-apoptotic effects are observed in LS174T cells upon exogenous FGF-BP transfection also suggests that a maximum threshold level of FGF-BP in LS174T cells is already reached by endogenous FGF-BP (over-)expression. Notably, the induction of apoptosis upon FGF-BP knockdown also coincides with the activation of cell death receptors TrailR1 and TrailR2 (DR4 and DR5, respectively) and, to a lesser extent, Fas/TNFR. This indicates apoptosis activation through the extrinsic pathway, and the Bax activation observed here suggests further signalling in a 'type II cell' manner (see [54] for review).

In FGF-BP knockdown cells, we also observed an imbalance in the redox status. FGF-BP depletion led to a modest decrease in catalase and modest increase in HIF1α levels. It has been shown previously that in a tumor, a number of compensatory mechanisms can occur under hypoxic conditions (see [55] for review). This includes an increased expression of pro-angiogenic growth factors leading to angiogenesis/neo-vascularization, and a cell matrix remodelling/increased heparan sulphate proteoglycan synthesis that leads to higher numbers of FGF2 binding sites and thus HIF1α-mediated increase in FGF signalling [56]. This effect is paralleled by an increase in NDST-1 activity [56] which in turn positively influences FGF-BP expression [57]. Taken together, this indicates that FGF-BP is involved in an autocrine regulation loop.

Additionally, alterations in Akt activity as shown above may also interfere with cellular sensitivity towards oxidative stress ('Akt-induces ROS-triggered cell death'). More specifically, Nogueira et al. showed that, upon Akt hyperactivation, cells are more prone to oxidative stress and intracellular accumulation of ROS through increased oxygen consumption and decreased expression of ROS scavengers [58]. Beyond the direct pro-apoptotic effects of ROS, this may be relevant for the sensitivity/resistance towards certain cytostatics acting through ROS generation. Notably, in our study decreased catalase levels were observed upon FGF-BP knockdown, suggesting impaired protection against oxidative stress. While this supports again the pro-apoptotic effect of FGF-BP inhibition, it also indicates that FGF-BP levels may determine the sensitivity of tumor cells towards chemotherapy. Indeed, this is observed for certain cytostatics dependent on their mechanism of action (Schulze et al., manuscript in preparation). Taken together, our data indicate that FGF-BP is integrated in a complex network of cytoprotective effects.

The therapeutic relevance of our findings is demonstrated by our in vivo data in mice. By employing siRNA-loaded nanocarriers for a therapeutic in vivo knockdown approach in established wildtype tumor xenografts, this study goes beyond previous publications based on ex vivo generated stable knockdown cell lines that were s.c. injected and thus do not mimick a therapeutic situation [29]. In vivo studies using FGF-BP-specific siRNAs have so far been limited to microinjection into chicken embryos in order to analyse the role of chBP in embryo development [59] or the use of morpholinos during zebrafish embryongenesis [60]. Here, however, we explore for the first time a therapeutic FGF-BP knockdown approach in tumors. To this end, we employ polymer-based nanoparticles which allow the in vivo delivery of siRNAs upon their systemic application ([35]; see [61] for review). Previous studies had demonstrated the absence of non-specific effects (acute toxicity, activation of immune responses, upregulation of inflammatory cytokines) of the PEI/siRNA nanoparticles [35]. In accordance with previous results [35], the determination of the levels of [^32^P]-labelled siRNAs demonstrate efficient delivery of intact siRNAs into the tumors. This is true even upon systemic administration which is more relevant in a therapeutic setting than local (intratumoral) injection. Concomitantly, a ~30% knockdown of FGF-BP expression is observed which proved sufficient for anti-tumor effects. This is in line with previous findings in stable FGF-BP knockdown prostate carcinoma cells, which showed an already complete abrogation of tumor formation upon injection of cells with 50% reduced FGF-BP levels [15], and further supports the possible relevance of FGF-BP as a therapeutic target molecule.

## Conclusions

Taken together, the knockdown of FGF-BP exerts multiple cellular and molecular effects in colon carcinoma including the induction of apoptosis, and FGF-BP represents a promising therapeutic target, for example by RNAi-based knockdown approaches through delivery of therapeutic siRNAs.

## List of Abbreviations

FGF-BP: Fibroblast growth factor-binding protein; PEI: polyethylenimine; RNAi: RNA interference; siRNA: small interfering RNA.

## Competing interests

The authors declare that they have no competing interests.

## Authors' contributions

DS generated the LS174T cell lines and analysed cellular and molecular effects of the FGF-BP knockdown (FGF-BP expression levels, proliferation assays, cell cycle measurements, induction of apoptosis, antibody arrays, Western blots); PP generated the HCT-116 and HT29 cell lines, analysed the cellular (proliferation assays including inhibitor, FGF2 rescue and HCT-116 p21-/- cell studies, induction of apoptosis) and molecular effects of the FGF-BP knockdown (Western blots); SH and DS performed the in vivo studies; AA conceived and coordinated the study, participated in the data analysis and wrote major parts of the paper. All authors read and approved the final manuscript.
